# Association between body weight and hip dysplasia screening results in young adult dogs of different breeds in Sweden

**DOI:** 10.1038/s41598-026-55816-y

**Published:** 2026-06-03

**Authors:** Linda Andersson, Karolina Engdahl, Sara Ringmark, Sofia Malm Persson, Charlotte Reinhard Bjørnvad, Åke Hedhammar, Katja Höglund

**Affiliations:** 1 Department of Animal Biosciences, Swedish University of Agricultural Sciences, Box 7023, Uppsala, 750 07 Sweden; 2 Department of Clinical Sciences, Swedish University of Agricultural Sciences, Box 7054, Uppsala, 750 07 Sweden; 3 Department for Breeding and Health, Swedish Kennel Club, Box 771, Sollentuna, 191 27 Sweden; 4 Department of Veterinary Clinical Sciences, University of Copenhagen, Dyrlaegevej 16, Frederiksberg C, 1870 Denmark

**Keywords:** Body weight, Hip dysplasia, Dog, Canine, Breed, Veterinary, Radiographic screening, Diseases, Genetics, Health care, Medical research, Zoology

## Abstract

Canine hip dysplasia (HD) is a common developmental orthopaedic disorder with substantial impact on health and life expectancy. Body weight has been considered an important factor for the development, but prior studies evaluating this association on a large data set, strictly collected and without preselection of screening results, are lacking. The objectives of the study were to investigate a potential association between body weight and radiographic HD screening grade in young adult dogs of different breeds, and to study potential breed differences in selected breeds of different sizes. A cross-sectional study using hip screening results and body weight data from the Swedish Kennel Club, collected during 2007–2016 from 114,568 young adult dogs of 72 different breeds in Sweden, was performed. An association between HD screening grade and body weight was found; higher body weight within breed was associated with a more severe HD grade. The association was breed-dependent and significant in 13 of the 21 breeds included in the analysis on breed differences. In conclusion, higher body weight within breed was significantly associated with a more severe HD screening grade, and the association was breed dependent. This indicates that lower body weight might be a protective factor for developing HD.

**Supplementary Information:**

The online version contains supplementary material available at 10.1038/s41598-026-55816-y.

## Introduction

Hip dysplasia (HD) is a common developmental orthopaedic disease in dogs^1,2^. It is considered a complex hereditary disorder with a genetic background, but environmental factors such as nutrition, rate of growth and types of exercise may affect the development and severity of the disease^3–7^. Hip dysplasia develops during the rapid phase of growth and although considered most common in large and giant-sized, fast-growing breeds, it can affect dogs of any breed and size^7–9^. A dysplastic hip exhibits varying degrees of poorly shaped joint and laxity, which can lead to instability, and over time, secondary osteoarthritis (OA) often develops^1,9,10^. Hip dysplasia can cause no or mild discomfort, but also severe pain and fulminant restriction of movement, leading to extensive need for rehabilitation and/or surgery or even early euthanasia^8,9,11^.

Radiographic hip joint assessment on dogs without clinical signs is performed within different screening programs in many countries, with the aim of being a breeding tool to reduce the prevalence of HD in the population^12,13^. The hip screening protocol used by the Swedish Kennel Club (SKC) has been developed by the Fédération Cynologique Internationale (FCI) and is available for all breeds from an age of 12 months (or 18 months for certain larger/giant breeds) for an official screening result^14,15^. Around 70% of the Swedish dog population is registered in the SKC, which is a substantial proportion compared to many other countries^16,17^. The proportion of dogs participating in the hip screening program run by the SKC varies, but is 30–60% in many of the registered breeds, and the majority of the dogs are screened close to the official minimum age^17^. For many breeds, screening is a requirement for registration of offspring in the SKC. All radiographs taken by the veterinary clinics performing the radiographic examinations are submitted to the SKC for evaluation by appointed scrutineers and the results are mandatorily registered in the SKC database and publicly available. This precludes selection bias, compared to screening programs in some other countries where submission of radiographs and results registration are voluntary^13,18–21^. Since 2005, recording of the dogs’ body weight (BW) at the time of screening is mandatory in the SKC screening program.

The reported HD prevalence in dogs differs largely depending on the investigated populations^18,22–24^. Several risk factors for HD have been evaluated in previous studies of which some, such as breed, sex and age at screening, have been associated with HD^2,3,19,24–27^. Factors related to the radiographic procedure, like positioning of the dog and sedation method induce a risk of misclassification bias to the HD grading^26–28^.

High BW due to large body size or high percentage of body fat is considered a risk factor for both HD and OA^11,29–34^. However, previous studies on BW and HD have used varying data sources: from different screening programs or animal hospitals and included dogs with and without clinical signs and with different HD classifications. The studies have often been performed on only one or a few breeds, on preselected screening results and on listed breed BW rather than on individually collected BW ^4,6,33–37^, which might impact the validity of the results. Studies evaluating the association between HD and BW within breed on large screening datasets, strictly collected and without preselection of results, are lacking.

The aims of the current study were to: (1) investigate the association between BW and HD screening grade in young adult dogs (12–30 months of age, depending on breed) of different breeds participating in the hip screening program in Sweden, and (2) investigate potential breed differences.

## Materials and methods

### Study population

A data file with 183,252 records on dogs of 304 breeds born between 2005 and 2015 was acquired from the SKC in October 2018. The file included HD screening data from 2005 to 2018 and information about each dog, including registration number, sex, date of birth, screening date, and BW at the time of screening. Individuals without a Swedish registration number, with results from a former grading system or screening performed before the officially accepted age (12 months for most breeds, 18 months for certain large/giant breeds), and individuals with missing or extreme BW (biologically implausible for the breed) were excluded^38,39^. Screening results from 2006 were excluded as the average age of the dogs at screening was lower than in subsequent years, due to the dataset consisting of dogs born from 2005. Screening results from 2017 to 2018 were also excluded as the average age of the dogs at screening was higher than in previous years, as the dataset included dogs born until 2015. The first result of an individual was used when more than one screening result was available. Young adult dogs, defined as having screening results at 12–24 months of age, or 18–30 months in certain large breeds (Table S1), in breeds with at least 15 screening results per year during ten years of screening (2007–2016), were included in the study population.

### Screening and grading of hip dysplasia

The SKC hip screening program for HD is performed according to the official protocols of FCI^14,15,40^. The dog must be anaesthetised or sufficiently sedated for full muscle relaxation during the radiographic screening procedure, according to the FCI protocol. The dog is positioned on its back with its hind legs extended during the radiographic examination. The radiographs are centrally evaluated by SKC scrutineers, and the assessment is based on the contour and shape of the hip joint, the fit between the acetabulum and the femoral head and the position of the femoral head inside the acetabulum. Furthermore, signs of OA (such as osteophyte formation) are noted. The hips are graded in five categories: HD grade A and HD grade B corresponding to non-dysplastic (normal) joints and HD grade C, HD grade D and HD grade E corresponding to mild, moderate and severe dysplasia, respectively^40^.

### Risk factors for hip dysplasia

Body weight was defined as the main exposure variable, and age at screening, mode of sedation, year of screening, breed, and sex were included as other potential risk factors for HD in the analysis. Age at screening and BW were standardised within breed to account for breed-related differences. This was done by subtracting the breed mean from the observed value and dividing by the breed-specific standard deviation. The variable for mode of sedation included two categories: one with dogs sedated with acepromazine used solely or combined with other sedative or analgesic drugs, and one with dogs sedated with substances other than acepromazine.

### Statistical analysis

R version 4.2.1 was used for the statistical analyses^41^. Categorical variables are presented as numbers and percentages per category and continuous variables as median (IQR, total range). One-proportion Z-test was used to compare proportions. Kruskal–Wallis one-way analysis of variance was used to compare BW between breeds, and Fisher’s exact test was used to evaluate the association between breed and HD screening grade. A p-value of ≤ 0.05 was considered statistically significant, unless otherwise indicated.

Ordinal logistic regression was used to model associations between the main exposure (BW), potential confounders, and HD screening grade (four categories: HD grade A, HD grade B, HD grade C and HD grades DE). Grades D and E were combined in the analysis due to the relatively few individuals in each group. Two separate models were built. The first model included age at screening, mode of sedation, year of screening, and sex as potential confounders. All variables with *P* ≤ 0.15 in univariable analyses were considered for inclusion in the multivariable models. Breed was included as a random effect in the multivariable model, while the other predictors were included as fixed effects. The clmm() function from the R-package ordinal was used to build the model^42^. A p-value of ≤ 0.001 was considered statistically significant in the multivariable model. The low p-value was chosen to minimise the risk of type I error^43^. Manual stepwise backwards elimination was applied for variable selection. The Wald test was used to evaluate the significance of multilevel categorical variables. The assumption of proportional odds was evaluated by running stratified binomial models and checking for similar coefficients of the effects.

A second model was used to compare the probability of a more severe grade of HD in a subset of breeds. A new variable was generated with separate categories for 21 breeds (Boxer, Chow Chow, Cocker Spaniel, Bernese Mountain Dog, Bullmastiff, Dogue de Bordeaux, Danish-Swedish Farmdog, Border Collie, English Springer Spaniel, American Staffordshire Terrier, Finnish Lapponian Dog, Lagotto Romagnolo, Newfoundland, Rottweiler, Samoyed, Portuguese Water Dog, St. Bernard, Welsh Springer Spaniel, Spanish Water Dog, Staffordshire Bull Terrier, and Tibetan Terrier). The breeds were selected based on size, including small, medium, large, and giant breeds, as well as HD screening grade distribution, aiming at including breeds with variation in size as well as HD grade. All other breeds were categorised as “Other”, and this category was used as the baseline in the analysis. The new breed variable was included as a fixed effect in the model in addition to those in the first model. The model-building process was similar to the first model and the same significance levels were used, but the clm() function was used instead of the clmm() function (as there were no random effects in the second model).

Biologically plausible interactions were considered in both models. A coefficient, standard error, and p-value was reported for each variable in the regression models. In addition, the threshold coefficients (i.e. cut-offs between the categories of the outcome) were reported. Interactions were explored with the emtrends() function from the emmeans package^44^ and plotted with plot_model() from the sjPlot package^45^. Interactions causing a violation of the assumption of proportional odds were excluded.

## Results

In total, 114,568 dogs (62.5%) from 72 breeds remained in the study population after applying the inclusion and exclusion criteria. The number of dogs per breed is presented in Table S1. Three of the 72 breeds (4.2%) were represented by > 10,000 individuals, 30 breeds (41.7%) by > 1,000 individuals, and 45 breeds (62.5%) by > 500 individuals. The median age at screening was 1.3 years (IQR 1.1–1.6, total range 1.0-2.5). Sixty-five breeds (90.3%) included dogs screened at 12–24 months of age, and the remaining seven breeds (9.7%) included dogs screened at 18–30 months of age (Table S1). In breeds screened between 12 and 24 months of age, the median age at screening was 1.3 years (IQR 1.1–1.5, total range 1.0–2.0), and in breeds screened between 18 and 30 months, the median age at screening was 1.8 years (IQR 1.6-2.0, total range 1.5–2.5). In the study population, the number of dogs screened yearly varied from 9,957 to 13,466 during the study period.

There were more females (*n* = 60,417) than males (*n* = 54,151) in the study population (52.7% and 47.3%, respectively, one-proportion Z-test, *P* < 0.001). The median BW was 27.0 kg (IQR 20.0–33.0, total range 3.4–91.0) and varied with breed (Kruskal–Wallis one-way analysis of variance, *P* < 0.001, Table S1). There were more dogs sedated with other drugs (*n* = 96,315, 84.1%) than with acepromazine solely or in combination with other drugs (*n* = 18,253, 15.9%, one proportion Z-test, *P* < 0.001).

The distribution of HD screening grades in the study population was as follows; 61,660 (53.8%) dogs had HD grade A, 30,222 (26.4%) had HD grade B, 15,957 (13.9%) had HD grade C, 5,863 (5.1%) had HD grade D, and 866 (0.8%) had HD grade E. Thus, the combined group of dogs with HD grades A and B (non-dysplastic hips) included 91,882 (80.2%) dogs and HD grades C, D and E (dysplastic hips) included 22,686 (19.8%) dogs. The combined group of dogs with HD grades D and E included 6,729 (5.9%) dogs. The percentage of dogs with each HD screening grade varied with breed (Fisher’s exact test, *P* < 0.001, Table S1).

The results from the first model are presented in Table 1. All variables were retained in the model, together with an interaction between age and mode of sedation. A higher BW within breed increased the probability of a more severe HD screening grade. A higher age within breed also increased the probability of a more severe HD grade. The probability of a more severe HD grade was also higher in females than in males, in dogs sedated with substances other than acepromazine, and increased with time (i.e. the year of screening). The increased probability of a more severe HD grade at a higher age was less pronounced in dogs sedated with other drugs than acepromazine.

**Table 1:** The association between body weight and hip dysplasia (HD) screening grade, controlling for potential confounders, in dogs participating in a hip screening program during 2007–2016. Results from an ordinal logistic regression model with the HD variable categorised as A, B, C, and DE, where A and B represent non-dysplastic hips and C and DE dysplastic hips.

Variable	Coefficient	Standard error	*P* value
**Body weight (standardised)**	0.19	0.01	< 0.001
**Age (standardised)**	0.19	0.02	< 0.001
**Mode of sedation**			< 0.001
Acepromazine	Baseline		
Other	0.36	0.02	
**Sex**			< 0.001
Male	Baseline		
Female	0.25	0.01	
**Year of screening**	< 0.01	< 0.01	< 0.001
Age (standardised)*mode of sedation (acepromazine)	Baseline		
Age (standardised)*mode of sedation (other)	-0.06	0.02	< 0.001
**Threshold coefficients**			
A|B	0.59	0.09	
B|C	1.96	0.09	
C|DE	3.41	0.10	
A random effect for breed	Variance	Standard deviation	
(72 levels)	0.61	0.78	

The results from the second model, focusing on breed-related differences, are presented in Table 2. All variables as well as interactions between breed and all other variables, except for the mode of sedation, were retained in the model. As the focus of this analysis was breed-related differences in the association between BW and HD screening grade, the interaction between breed and BW is presented in Table 2, while the other interactions are presented in Table S2. The coefficients for sex, age, BW, and mode of sedation were relatively similar to the first model. The coefficient for the year of screening changed from positive to negative, and the main effect was no longer significant, although the interaction between breed and age of screening indicated that the association between the year of screening and HD grade was breed-dependent. Note that in the results presented in Table 2, each breed is compared to the “Other” category, which serves as the baseline in the analysis and consists of all other breeds in the study population than the ones included as separate categories.

**Table 2:** The results from an ordinal logistic regression model evaluating the association between body weight and hip dysplasia screening grade, with special focus on breed-related differences, in dogs participating in a hip screening program during 2007–2016. The variable for breed consists of 21 separate breeds, as well as the category “other”, including dogs of all remaining breeds used as the baseline (*n* = 51). The interaction between body weight and breed is presented in this table, while interactions between breed and sex, age, and year of screening are presented in Table S2.

Variable	Coefficient	Standard error	*P* value
**Body weight (standardised)**	0.18	0.01	< 0.001
**Age (standardised)**	0.12	< 0.01	< 0.001
**Breed**			< 0.001
Other	Baseline		
American Staffordshire Terrier	2.02	0.14	< 0.001
Bernese Mountain Dog	-0.15	0.08	0.055
Border Collie	<-0.01	0.10	0.969
Boxer	0.89	0.09	< 0.001
Bullmastiff	2.27	0.23	< 0.001
Chow Chow	-0.12	0.24	0.608
Cocker Spaniel	0.24	0.10	0.016
Danish-Swedish Farmdog	0.18	0.12	0.118
Dogue de Bordeaux	2.67	0.24	< 0.001
English Springer Spaniel	-0.35	0.12	0.003
Finnish Lapponian Dog	0.73	0.12	< 0.001
Lagotto Romagnolo	0.85	0.10	< 0.001
Newfoundland	1.10	0.21	< 0.001
Portuguese Water Dog	0.42	0.15	0.004
Rottweiler	-0.52	0.07	< 0.001
Samoyed	-0.02	0.14	0.881
Spanish Water Dog	0.25	0.16	0.112
St. Bernard	2.20	0.25	< 0.001
Staffordshire Bull Terrier	1.53	0.12	< 0.001
Tibetan Terrier	-0.17	0.21	0.414
Welsh Springer Spaniel	0.41	0.11	< 0.001
**Mode of sedation**			< 0.001
Acepromazine	Baseline		
Other	0.27	0.02	
**Sex**			
Male	Baseline		
Female	0.30	0.02	< 0.001
**Year of screening**	<-0.01	< 0.01	0.278
Body weight (standardised) * breed			< 0.001
Body weight (standardised) * Other	Baseline		
Body weight (standardised) * American Staffordshire Terrier	-0.01	0.07	0.864
Body weight (standardised within breed) * Bernese Mountain Dog	0.13	0.04	0.001
Body weight (standardised) * Border Collie	-0.06	0.05	0.208
Body weight (standardised) * Boxer	-0.26	0.05	< 0.001
Body weight (standardised) * Bullmastiff	-0.17	0.12	0.174
Body weight (standardised) * Chow Chow	0.01	0.10	0.910
Body weight (standardised) * Cocker Spaniel	-0.24	0.05	< 0.001
Body weight (standardised) * Danish-Swedish Farmdog	0.06	0.05	0.246
Body weight (standardised) * Dogue de Bordeaux	-0.08	0.12	0.473
Body weight (standardised) * English Springer Spaniel	-0.21	0.06	< 0.001
Body weight (standardised) * Finnish Lapponian Dog	-0.06	0.05	0.268
Body weight (standardised) * Lagotto Romagnolo	0.14	0.05	0.002
Body weight (standardised) * Newfoundland	0.03	0.10	0.742
Body weight (standardised) * Portuguese Water Dog	0.02	0.08	0.793
Body weight (standardised) * Rottweiler	0.09	0.04	0.023
Body weight (standardised) * Samoyed	< 0.01	0.07	0.923
Body weight (standardised) * Spanish Water dog	0.30	0.07	< 0.001
Body weight (standardised) * St. Bernard	-0.03	0.12	0.777
Body weight (standardised) * Staffordshire Bull Terrier	0.07	0.05	0.159
Body weight (standardised) * Tibetan Terrier	-0.05	0.11	0.673
Body weight (standardised) * Welsh Springer Spaniel	-0.01	0.06	0.874
**Threshold coefficients**			
A|B	0.62	0.02	
B|C	1.93	0.02	
C|DE	3.37	0.03	

**Fig. 1:**
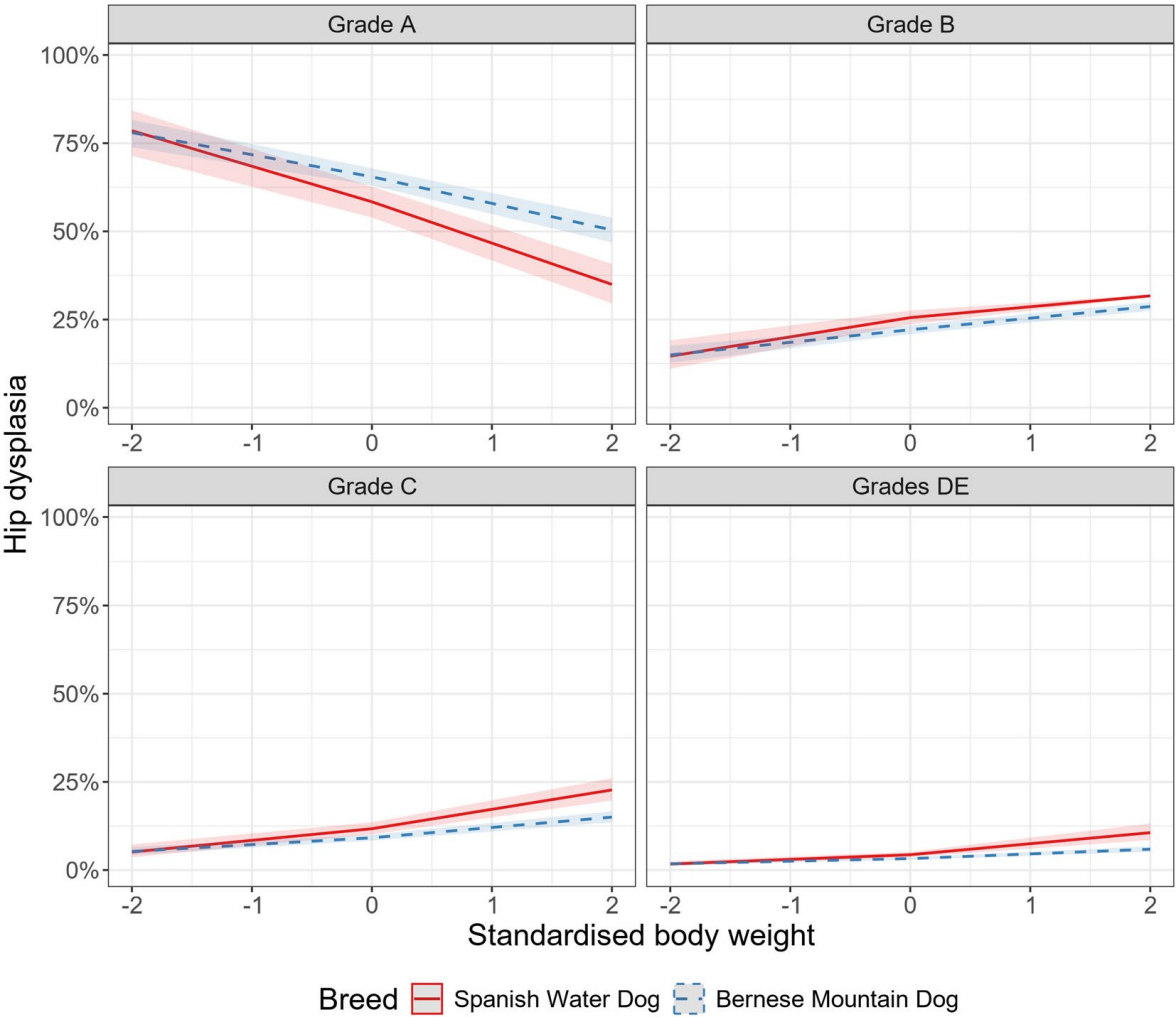
Predicted probabilities of hip dysplasia (HD) screening grades A, B, C, and DE in the Bernese Mountain Dog and Spanish Water Dog for different body weights (BW, standardised within breed), based on the results presented in Table 3. The association between BW and HD grade was significant in both breeds and an increased BW was associated with an increased probability of more severe HD. The y-axis shows the predicted probability of each grade (A, B, C and DE) and the x-axis shows the BW standardised within breed as the breed mean BW at 0 and 1 and 2 standard deviations from the breed mean BW.

The interaction between breed and BW was further explored, and the coefficients/trends for the association between BW and HD screening grade within each breed are presented in Table 3. Breeds with an increasing probability of a more severe HD grade with increasing BW have a positive coefficient/trend, with a confidence interval that does not include zero. In contrast, breeds with no significant association between BW and HD grade have confidence intervals that include zero. Figure 1 shows examples of predicted probabilities for different HD grades depending on BW (standardised within breed) in the Bernese Mountain Dog and the Spanish Water Dog.

**Table 3:** The linear relationship between body weight and the probability of a more severe hip dysplasia screening grade (shown as the trend of the slope) within each breed, based on the model presented in Table 2. In total, 21 separate breeds were included, together with the category “other”, including dogs of all remaining breeds (*n* = 51). Breeds with a significant association between body weight and hip dysplasia are shown in bold.

Breed	Body weight (standardised), trend	Standard error	Confidence interval (for trend)
**American Staffordshire Terrier**	0.17	0.06	0.05–0.30
**Bernese Mountain Dog**	0.31	0.04	0.24–0.39
**Border Collie**	0.12	0.05	0.02–0.22
Boxer	-0.07	0.05	-0.17–0.03
Bullmastiff	0.02	0.12	-0.22–0.26
Chow Chow	0.20	0.10	-0.01–0.40
Cocker Spaniel	-0.06	0.05	-0.15–0.04
**Danish-Swedish Farmdog**	0.25	0.05	0.14–0.35
Dogue de Bordeaux	0.10	0.11	-0.12–0.33
English Springer Spaniel	-0.03	0.06	-0.14–0.08
**Finnish Lapponian Dog**	0.13	0.05	0.03–0.23
**Lagotto Romagnolo**	0.32	0.04	0.23–0.41
**Newfoundland**	0.22	0.10	0.02–0.41
**Other**	0.18	0.01	0.17–0.20
**Portuguese Water Dog**	0.20	0.08	0.06–0.35
**Rottweiler**	0.27	0.04	0.20–0.34
**Samoyed**	0.19	0.07	0.06–0.33
**Spanish Water Dog**	0.48	0.07	0.35–0.61
St. Bernard	0.15	0.12	-0.09–0.39
**Staffordshire Bull Terrier**	0.25	0.05	0.16–0.35
Tibetan Terrier	0.14	0.10	-0.07–0.35
**Welsh Springer Spaniel**	0.18	0.06	0.07–0.28

## Discussion

This study on 114,568 dogs from 72 breeds evaluated the association between BW and HD screening grade in dogs participating in a hip screening program in Sweden, with a specific focus on breed-related differences. In general, the probability of a more severe HD grade increased with increasing BW within breed. Breed-related differences in the association between BW and HD grade were also explored and revealed the BW association with HD grade to be breed dependent, with a significant association in the majority of included breeds. Although several previous studies on the association between BW and HD exist, this is to our knowledge the first study investigating the association between adult BW and HD screening grade within breed in a large number of breeds^33^.

Some prior studies on BW and HD have focused on birth weight and BW early in life, as well as on growth rate and the associated weight gain^5,7,9,10,46,47^. During the fragile development of the hip joint, the joint stability depends mainly on the surrounding soft tissue. High BW in early life can potentially and negatively affect this, and thus also the laxity of the joint, which is important for the development of HD^48,49^.

Similar to the present study, other studies on BW and HD have used adult BW, but with different study designs. In one study, including two groups of Labrador Retrievers which were fed either limited or unlimited food during growth, the limited-fed dogs were found to have less severe HD than the other group, possibly due to a lower BW at adulthood^6^. In two other studies, the BWs of different breeds were analysed with hip screening results^34,36^. An association between breeds with higher BW and the prevalence of HD was found. However, these studies are limited by using listed breed BW instead of BW from individual dogs, and analyses were performed per breed and not within breed. Furthermore, the HD data used was derived from screening programs with voluntary submission and recording of screening results.

In the present study, a separate analysis was performed on the association between HD screening grade and BW within breed in 21 different breeds. A significant association was found in 13 of the 21 breeds, where the probability of a more severe HD screening grade increased with increasing BW within breed (Table 3). The effect was most evident in the Spanish Water dog, Lagotto Romagnolo and Bernese Mountain Dog. In the Spanish Water Dog, the predicted probability of HD grade A for a dog with a BW two standard deviations lower than the breed mean was around 80%, as illustrated in Figure 1. The corresponding probability for a dog of the same breed weighing two standard deviations more than the breed mean was around 30%. The increasing probability of a higher HD grade with increasing BW was also evident in the Portuguese Water Dog, a breed closely related to the Spanish Water Dog and Lagotto Romagnolo. The latter three are all popular breeds, with similar body type and size, which were originally used as working dogs but are nowadays mostly companion dogs. All in all, the breeds with the significant association between BW and HD grade are of different types and uses, from the larger Bernese Mountain Dog and Newfoundland to the smaller Danish-Swedish Farmdog, and do not all share obvious common traits. Several different sizes being represented suggests that BW may play an important role also in smaller-sized breeds, where HD is not as common as in larger-sized breeds. In a few breeds, however, no association between BW and HD grade was found, and these breeds show the same diversity in size and use. It is possible that BW is not as important a factor for HD development in these breeds compared to others, but the reason for this is unknown. Prior research has shown that body conformation, like muscle mass and body shape, could be associated with HD, and that the same amount of laxity could be tolerated differently in different breeds before HD-related degenerative changes occur^36,37,50,51^. Such factors or others, unknown to us, might have played a greater role than the BW in these breeds and affected the association between BW and HD grade in the current study. It is also possible that the genetic influence has been of greater importance than BW in these breeds, as HD is a quantitative trait with varying heritability estimates in different breeds^2,3,24^. Another possibility is that the size of the study population may have affected the result. For example, the majority (12 out of 13) of breeds that had an association between HD and BW included 1000 or more dogs, whereas five of the eight breeds without an association were represented by less than 500 dogs. Although 500 dogs is a large number, this might have decreased the statistical power.

The variation in HD prevalence between breeds in the current study is high (Table S1), as reported earlier in a study performed on the same dataset^52^ and in other studies^18,19,23^. Eleven of the 13 breeds with a significant association between BW and HD grade in the current study had a HD prevalence (HD grades C, D and E) over 19% and five of those breeds had a prevalence over 30% (Table S1). The high prevalence of HD in many of the breeds with a significant association between HD grade and BW in the present study is discouraging, since the effect of HD on overall health, wellbeing and lifespan of the dogs could be substantial^5,8,11,53,54^. The clinical signs of HD might develop early, but more severe signs are often seen if OA develops and progresses in older dogs^1,5,8,11,53,54^. Body weight also plays a role in OA development. The laxity in the joint creates incongruence, altered movement, abnormal cartilage wear, and inflammation^1,9,10,37,55–58^. This process can be affected by a higher BW depending on a larger size or greater amount of fat, through additional load on the joints and joint inflammation induced by hormones secreted by the adipose tissue^58–60^.

Body weight in early adulthood was the only available weight estimate for dogs included in this study and no other body measurements were available. Therefore, high BW could depend on a larger size, a high muscle mass or more fat. However, regardless of whether the association between BW and HD grade found in this study depends on size or body fat, the clinical significance of the association is important to address. For example, in the breed standards for some breeds, such as the Dogue de Bordeaux, Newfoundland, Bernese Mountain Dog and Bullmastiff, descriptions such as stocky, massive, imposing, sturdy, compact and robust are used to describe the ideal shape and size. Additionally, in some breeds, the breed BW is not given as a range but as a minimum^61^. These wordings and goals could be problematic and conflict with the dog’s health if exaggerated. Although efforts have been made to favour the breeding of sound and healthy dogs, exaggerations in size and BW are still present in some breeds, of which some had an association between HD grade and BW in this study^62^. Furthermore, the reported prevalence of overweight and obesity in dogs is high nationally^63^ and internationally and appears to be increasing^64–71^. Some breeds are more prone to obesity than others, and its effect on the overall dog’s health, including on HD, could be significant^65,70–73^. In the study on BW and BW changes over time on the same data, an increase in BW was observed in some breeds, for example in the Bernese Mountain Dog^39^. This is not beneficial, given both their high prevalence of HD and the identified association between HD grade and BW in the current study.

Overall, the within-breed association between HD screening grade and BW found in the present study highlights the importance of preventing overweight and obesity, regardless of breed size, and of not encouraging breeding for increased size and shape. Further studies on HD and BW, including repeated measurements of BW, and with other body measurements such as body condition score and height included, would be beneficial to determine the background of the association.

Some additional risk factors for a more severe HD screening grade were identified in the current study. Female dogs had a higher probability of a more severe HD screening grade than male dogs. This is in line with other studies using HD screening data in Sweden^2,3,26,74^, and from other countries^27,75–77^. This might depend on differences in sex hormones and growth rate^2,39,78–80^. The higher risk of overweight in female dogs than in male dogs might furthermore affect the association^64,65,81–84^. Being neutered has been identified as a risk factor for HD due to weight gain after neutering and hormonal effects on joints^85,86^. Information about neutering status was not available in the current study, but neutering is fairly uncommon in young healthy dogs in Sweden, and thus the effect is likely of lesser significance.

An association between higher age and HD has been shown in previous studies on screening data in Sweden^2,26^, as well as studies from other countries on single breeds^25,27,77,87^. This has been explained by the fact that OA changes tend to get worse with age. Interestingly, despite the narrow age span of 12–24 months (18–30 months in some large breeds), higher age at screening increased the probability of more severe HD grade, also in the present study. Furthermore, in the FCI scheme used in the SKC hip screening program, OA might be present in HD grade C, D and E. Presence of OA is noted separately, but does not differentiate between the grades of dysplasia, which is not the situation in all national screening programs^13,40^.

Screening year was also associated with HD grade, and in general, the probability of a more severe HD grade increased during the study time, although the magnitude of this effect was low. This might depend on different reasons. One possible explanation is the recent transition from analogue to digital radiographic systems, and thereby easier detection of subtle abnormalities in the radiographs.

Dogs sedated with substances other than the neuroleptic drug acepromazine had a higher probability of a more severe HD screening grade than dogs sedated with acepromazine solely or combined with other substances, in line with findings in a previous study^26^. Acepromazine provides a lighter sedation and less muscle relaxation than alpha-2 agonists, another commonly used drug^88^. It is suspected that insufficient sedation and relaxation might mask joint laxity and affect the grading of the hips^17,89–91^. In contrast to the FCI hip screening protocol used in Sweden, which requires sedation or anaesthesia, this is not mandatory in all national screening programs^13^. The association was less pronounced in older dogs within the age span of 12–24/18–30 months, depending on breed (age was standardised within breed in the analysis). Both high BW and different tendencies to hip joint laxity have previously been pointed out as possible factors that can affect how the different sedative drugs affect laxity and HD^26^. One could furthermore expect that older dogs have better joint stability due to more muscle mass but also less elastic muscles than younger dogs^92^. Hence, even with full sedation with an alpha-2 agonist, the joint may remain tighter in an older dog than in a younger dog and give rise to less of a difference in the association compared to sedation with the less potent acepromazine.

Some important aspects should be considered regarding the generalisability and interpretation of the results of this study. The data used in this study differs from the data used in some other studies on BW and HD, which affects the comparability of the results. Hip dysplasia screening programs have different rules and regulations regarding age, sedation method, submission and registration of screening results, as well as grading of HD^13^. Studies based on data from veterinary hospitals might include animals with clinical signs and thus more severely affected dogs compared to data from screening programs. Due to the inclusion and exclusion criteria, older dogs are not represented in the study. Smaller-sized breeds are underrepresented based on the low numbers of dogs participating in screening in those breeds. However, the size of the data and the large proportion of Swedish dogs registered in SKC and participating in the screening entail that the included breeds and age groups are representative of the general Swedish dog population. Since dogs with clinical signs related to their hips before 12 months of age are unlikely to participate in screening to the same extent, severe cases of HD are probably underrepresented. The use of different sedatives might differ between the veterinary clinics performing HD screening, and the mode of sedation has therefore been adjusted for in the model. Due to the retrospective nature of the study, the causality of the association between BW and HD screening grade cannot be confirmed, as well as the direction of the association, as BW at screening was the only available BW. A p-value limit of 0.001 was used in the regression models to decrease the risk of type one errors. However, this instead increased the risk of type two errors, which should be considered when the results are interpreted. As a consequence of a lower number of individuals in some dog breeds, type two error could not be excluded in the analyses on breed differences.

In conclusion, a higher BW within breed was associated with more severe HD grade at radiographic screening in this study on a large number of young adult dogs of different breeds in Sweden. The association was breed-dependent and was present in both larger and smaller-sized breeds. The findings have clinical implications, as HD is a common locomotor disease and might lead to diminished health and a shorter lifespan in affected dogs. Lower BW could be a protective factor for the development of HD. Further studies on HD, body condition and BW over time would be beneficial to establish the causality of the association between BW and the severity of HD.

## Supplementary Information

Below is the link to the electronic supplementary material.

Supplementary Material 1

## Data Availability

The data that support the findings of this study are available from the Swedish Kennel Club, but restrictions apply to the availability of these data, which were used under approval, and so are not publicly available. Data are however available from the corresponding author (linda.andersson@slu.se) upon reasonable request and with permission from the Swedish Kennel Club.

## Abbreviations

HD: Hip dysplasia
BW: Body weight
SKC: Swedish Kennel Club
FCI: Fédération Cynologique Internationale
OA: Osteoarthritis
