# Supplementary material for: Association between body weight and hip dysplasia screening results in young adult dogs of different breeds in Sweden
Source: Sci Rep. 2026 Jun 3;16:17144. doi: 10.1038/s41598-026-55816-y (PMC13234431; doi:10.1038/s41598-026-55816-y)
Supplement: Supplementary file 1 — Supplementary Material 1 [file 41598_2026_55816_MOESM1_ESM.docx]

Supplementary tables

Table S1. The number of individuals per breed, body weight, and hip dysplasia (HD) screening grade at hip radiographic screening during 2007-2016, in a study on the association between body weight and HD grade in dogs. Dogs in most breeds were 12–24 months of age at screening, except for dogs in breeds marked with *, which were 18–30 months old at screening, according to official screening regulations. For each breed, body weight is presented as median (IQR, total range) and HD grade as the number of individuals and percentage per category (HD grade A and B corresponding to non-dysplastic (normal) joints and HD grade C, D and E corresponding to mild, moderate and severe dysplasia, respectively).

| **Breed** | **Number of dogs** | **Body weight in kg (IQR, total range)** | **HD grade A (%)** | **HD grade B (%)** | **HD grade C (%)** | **HD grade DE (%)** |
| --- | --- | --- | --- | --- | --- | --- |
| Alaskan Malamute | 343 | 34.0 (30.0-38.0, 20.0-52.0) | 205 (59.8) | 80 (23.3) | 41 (12.0) | 17 (5.0) |
| American Staffordshire Terrier | 1207 | 25.0 (22.5-28.0, 11.0-40.0) | 96 (8.0) | 336 (27.8) | 527 (43.7) | 248 (20.6) |
| Australian Kelpie | 839 | 17.0 (15.1-19.0, 10.0-28.0) | 484 (57.7) | 278 (33.1) | 70 (8.3) | 7 (0.8) |
| Australian Shepherd | 1867 | 21.0 (18.9-24.0, 12.0-36.8) | 1172 (62.8) | 509 (27.3) | 143 (7.7) | 43 (2.3) |
| Bearded Collie | 725 | 21.0 (19.2-23.5, 9.0-32.8) | 443 (61.1) | 191 (26.3) | 66 (9.1) | 25 (3.5) |
| Belgian Shepherd Dog, Groenendael | 364 | 21.0 (18.8-24.0, 12.0-35.0) | 243 (66.8) | 89 (24.5) | 24 (6.6) | 8 (2.2) |
| Belgian Shepherd Dog, Malinois | 1235 | 26.0 (23.3-30.0, 14.0-39.5) | 873 (70.7) | 251 (20.3) | 96 (7.8) | 15 (1.2) |
| Belgian Shepherd Dog, Tervueren | 656 | 22.4 (20.0-25.0, 14.6-40.0) | 474 (72.3) | 125 (19.1) | 42 (6.4) | 15 (2.3) |
| Bernese Mountain Dog | 3921 | 41.8 (38.0-46.0, 18.0-68.0) | 2264 (57.7) | 878 (22.4) | 457 (11.7) | 322 (8.2) |
| Border Collie | 2831 | 17.0 (15.0-19.0, 9.2-26.0) | 1624 (57.4) | 813 (28.7) | 327 (11.6) | 67 (2.4) |
| Boxer | 2563 | 28.0 (25.2-31.0, 17.0-44.5) | 962 (37.5) | 855 (33.4) | 623 (24.3) | 123 (4.8) |
| Briard | 674 | 32.0 (28.0-36.0, 20.0-51.7) | 355 (52.7) | 145 (21.5) | 108 (16.0) | 66 (9.8) |
| Brittany Spaniel | 229 | 15.0 (13.7-17.0, 10.1-30.0) | 66 (28.8) | 73 (31.9) | 62 (27.1) | 28 (12.2) |
| Bullmastiff* | 355 | 49.0 (44.6-54.0, 23.0-70.4) | 47 (13.2) | 77 (21.7) | 143 (40.3) | 88 (24.8) |
| Cane Corso | 659 | 41.2 (37.2-47.0, 27.0-76.5) | 237 (36.0) | 167 (25.3) | 141 (21.4) | 114 (17.3) |
| Chow Chow | 490 | 25.0 (23.0-27.5, 8.2-35.6) | 233 (47.6) | 88 (18.0) | 82 (16.7) | 87 (17.8) |
| Cocker Spaniel | 2295 | 12.4 (11.0-14.0, 8.0-27.6) | 1265 (55.1) | 672 (29.3) | 298 (13.0) | 60 (2.6) |
| Collie, short-haired | 348 | 22.9 (20.0-25.4, 14.9-36.6) | 306 (87.9) | 35 (10.1) | 6 (1.7) | 1 (0.29) |
| Collie, long-haired | 2076 | 22.0 (19.7-24.5, 13.0-43.3) | 1367 (65.9) | 407 (19.6) | 177 (8.5) | 125 (6.0) |
| Dalmatian | 300 | 28.1 (26.0-32.0, 19.0-40.0) | 172 (57.3) | 90 (30.0) | 33 (11.0) | 5 (1.7) |
| Danish-Swedish Farmdog | 1767 | 7.9 (6.8-9.0, 4.0-16.1) | 813 (46.0) | 572 (32.4) | 301 (17.0) | 81 (4.6) |
| Dobermann | 945 | 33.5 (30.0-37.4, 23.0-50.6) | 435 (46.0) | 305 (32.3) | 174 (18.4) | 31 (3.3) |
| Dogue de Bordeaux* | 334 | 51.3 (47.0-57.0, 35.0-75.0) | 32 (9.6) | 50 (15.0) | 109 (32.6) | 143 (42.8) |
| East Siberian Laïka | 436 | 24.0 (22.0-27.9, 16.0-38.0) | 361 (82.8) | 63 (14.5) | 12 (2.8) | 0 (0) |
| English Pointer | 305 | 21.0 (19.0-23.0, 12.9-33.4) | 209 (68.5) | 64 (21.0) | 25 (8.2) | 7 (2.3) |
| English Springer Spaniel | 1943 | 20.6 (18.9-23.0, 10.2-37.7) | 1270 (65.4) | 438 (22.5) | 191 (9.8) | 44 (2.3) |
| Eurasier | 694 | 23.0 (21.0-25.0, 13.6-37.1) | 431 (62.1) | 163 (23.5) | 64 (9.2) | 36 (5.2) |
| Finnish Hound | 412 | 24.0 (22.0-27.0, 16.4-38.0) | 189 (45.9) | 148 (35.9) | 55 (13.4) | 20 (4.9) |
| Finnish Lapponian Dog | 1577 | 16.0 (14.0-18.0, 8.8-28.6) | 557 (35.3) | 536 (34.0) | 397 (25.2) | 87 (5.5) |
| Flat-coated Retriever | 4710 | 29.0 (26.0-32.0, 17.0-43.8) | 3530 (75.0) | 850 (18.1) | 276 (5.9) | 54 (1.2) |
| German Shepherd Dog | 12335 | 31.6 (28.5-25.0, 15.0-60.0) | 4876 (39.5) | 4305 (34.9) | 2175 (17.6) | 979 (7.9) |
| German Spaniel | 1852 | 20.0 (18.0-22.7, 8.0-31.0) | 1143 (61.7) | 481 (26.0) | 154 (8.3) | 74 (4.0) |
| German Short-Haired Pointing Dog | 725 | 26.5 (23.6-29.3, 17.0-37.5) | 594 (81.9) | 97 (13.4) | 28 (3.9) | 6 (0.8) |
| German Wire-Haired Pointing Dog | 567 | 27.0 (24.0-29.5, 18.0-40.0) | 359 (63.3) | 122 (21.5) | 72 (12.7) | 14 (2.5) |
| Golden Retriever | 10960 | 30.0 (27.0-33.0, 15.0-52.7) | 4596 (41.9) | 3607 (32.9) | 1866 (17.0) | 891 (8.1) |
| Gordon Setter | 278 | 23.0 (19.6-26.0, 14.0-38.5) | 121 (43.5) | 110 (39.6) | 33 (11.9) | 14 (5.0) |
| Great Dane* | 398 | 62.0 (55.6-68.0, 43.0-88.0) | 194 (48.7) | 105 (26.4) | 85 (21.4) | 14 (3.5) |
| Hamilton Hound | 286 | 22.5 (20.4-25.2, 14.0-33.0) | 118 (41.3) | 111 (38.8) | 49 (17.1) | 8 (2.8) |
| Hovawart | 880 | 33.0 (30.0-36.8, 22.0-51.0) | 617 (70.1) | 158 (18.0) | 81 (9.2) | 24 (2.7) |
| Icelandic Sheepdog | 218 | 14.0 (12.0-15.5, 9.0-24.0) | 110 (50.5) | 56 (25.7) | 38 (17.4) | 14 (6.4) |
| Irish Soft-Coated Wheaten Terrier | 554 | 15.8 (14.2-17.0, 9.5-28.0) | 215 (38.8) | 221 (39.9) | 86 (15.5) | 32 (5.8) |
| Irish Red Setter | 1112 | 26.0 (22.6-29.5, 14.0-43.0) | 402 (36.2) | 409 (36.8) | 206 (18.5) | 95 (8.5) |
| Jämthund | 3884 | 25.0 (23.0-28.0, 15.0-41.5) | 2816 (72.5) | 716 (18.4) | 261 (6.7) | 91 (2.3) |
| Keeshond | 188 | 15.5 (14.0-17.6, 10.5-24.5) | 57 (30.3) | 74 (39.4) | 48 (25.5) | 9 (4.8) |
| Labrador Retriever | 13125 | 28.5 (25.0-32.0, 14.0-53.3) | 8233 (62.7) | 2771 (21.1) | 1488 (11.3) | 633 (4.8) |
| Lagotto Romagnolo | 2448 | 14.0 (12.5-16.0, 7.6-24.5) | 952 (38.9) | 758 (31.0) | 558 (22.8) | 180 (7.4) |
| Landseer* | 259 | 49.8 (45.0-54.0, (33.4-77.0) | 155 (59.9) | 50 (19.3) | 29 (11.2) | 25 (9.7) |
| Leonberger* | 1360 | 50.0 (45.0-55.0, 32.0-79.9) | 859 (63.2) | 219 (16.1) | 169 (12.4) | 113 (8.3) |
| Newfoundland* | 504 | 55.0 (49.0-60,0, 28.5-81.0) | 187 (37.1) | 110 (21.8) | 83 (16.5) | 124 (24.6) |
| Norwegian Elkhound, grey | 1629 | 18.0 (16.0-20.0, 10.5-30.0) | 1036 (63.6) | 389 (23.9) | 172 (10.6) | 32 (2.0) |
| Nova Scotia Duck Tolling Retriever | 2165 | 18.0 (16.0-20.0, 6.0-34.0) | 1280 (59.1) | 613 (28.3) | 215 (9.9) | 57 (2.6) |
| Portuguese Water Dog | 1000 | 20.1 (18.0-23.0, 12.2-35.0) | 491 (49.1) | 303 (30.3) | 150 (15.0) | 56 (5.6) |
| Pumi | 379 | 11.0 (9.7-12.0, 6.0-28.8) | 203 (53.6) | 117 (30.9) | 44 (11.6) | 15 (4.0) |
| Rhodesian Ridgeback | 2039 | 38.0 (34.0-42.0, 13.4-58.0) | 1523 (74.7) | 335 (16.4) | 131 (6.4) | 50 (2.5) |
| Riesenschnauzer | 720 | 34.0 (30.0-38.0, 20.8-52.0) | 409 (56.8) | 189 (26.3) | 89 (12.4) | 33 (4.6) |
| Rottweiler | 5994 | 39.8 (35.8-44.0, 23.3-62.7) | 4047 (67.5) | 869 (14.5) | 608 (10.1) | 470 (7.8) |
| Saint Bernard Dog, long-haired* | 317 | 64.0 (58.0-70.0, 40.0-91.0) | 90 (28.4) | 63 (19.9) | 82 (25.9) | 82 (25.9) |
| Samoyed | 1097 | 21.4 (19.0-24.0, 11.1-34.0) | 530 (48.3) | 319 (29.1) | 181 (16.5) | 67 (6.1) |
| Schapendoes | 298 | 15.0 (13.0-17.0, 9.3-24.0) | 207 (69.5) | 76 (25.5) | 15 (5.0) | 0 (0) |
| Shetland Sheepdog | 651 | 7.7 (6.5-9.2, 3.4-19.5) | 356 (54.7) | 193 (29.7) | 74 (11.4) | 28 (4.3) |
| Shiba | 373 | 10.0 (8.9-11.1. 6.0-17.3) | 221 (59.3) | 100 (26.8) | 33 (8.9) | 19 (5.1) |
| Small Münsterländer | 441 | 20.5 (18.5-23.0, 13.8-29.5) | 355 (80.5) | 57 (12.9) | 21 (4.8) | 8 (1.8) |
| Spanish Waterdog | 1163 | 17.0 (15.0-19.0, 10.0-28.0) | 561 (48.2) | 331 (28.5) | 183 (15.7) | 88 (7.6) |
| Stabijhoun | 325 | 20.0 (18.0-22.6, 12.0-29.0) | 95 (29.2) | 140 (43.1) | 74 (22.8) | 16 (4.9) |
| Staffordshire Bull Terrier | 2109 | 16.0 (14.4-17.8, 9.0-26.3) | 495 (23.5) | 731 (34.7) | 695 (33.0) | 188 (8.9) |
| Standard Poodle | 1149 | 20.5 (18.5-23.0, 12.5-36.0) | 687 (59.8) | 320 (27.9) | 114 (9.9) | 28 (2.4) |
| Swedish Lapphund | 313 | 15.8 (14.3-17.6, 9.8-32.5) | 114 (36.4) | 120 (38.3) | 60 (19.2) | 19 (6.1) |
| Swedish Vallhund | 416 | 12.0 (11.0-13.0. 7.5-17.5) | 159 (38.2) | 164 (39.4) | 79 (19.0) | 14 (3.4) |
| Tibetan Terrier | 461 | 10.3 (9.0-11.7, 5.9-17.8) | 264 (57.3) | 148 (32.1) | 34 (7.4) | 15 (3.3) |
| Welsh Springer Spaniel | 1634 | 17.8 (16.0-19.6, 11.3-28.8) | 760 (46.5) | 562 (34.4) | 210 (12.9) | 102 (6.2) |
| White Swiss Shepherd Dog | 606 | 30.5 (27.7-34.0, 20.0-47.0) | 336 (55.5) | 165 (27.2) | 82 (13.5) | 23 (3.8) |
| Working Kelpie | 256 | 18.3 (16.0-20.8, 12.0-29.0) | 122 (47.7) | 90 (35.2) | 32 (12.5) | 12 (4.7) |

Table S2. The interactions between breed and sex, age, and year of screening from model two, an ordinal logistic regression model evaluating the association between body weight and grade of hip dysplasia, controlling for potential confounders, with special focus on breed-related differences. The breed variable has separate categories for 21 breeds, and the “other” category consists of all other breeds (n=51) screened for hip dysplasia during the same period (2007-2016). The result of the rest of the model is presented in Table 2.

| **Interaction** | **Coefficient** | **Standard error** | **P value** |
| --- | --- | --- | --- |
| Age (standardised) * American Staffordshire Terrier | -0.05 | 0.05 | 0.332 |
| Age (standardised) * Bernese Mountain Dog | 0.07 | 0.03 | 0.038 |
| Age (standardised) * Border Collie | 0.04 | 0.04 | 0.278 |
| Age (standardised) * Boxer | 0.08 | 0.04 | 0.025 |
| Age (standardised) * Bullmastiff | -0.01 | 0.10 | 0.915 |
| Age (standardised) * Chow Chow | -0.01 | 0.09 | 0.876 |
| Age (standardised) * Cocker Spaniel | 0.11 | 0.04 | 0.010 |
| Age (standardised) * Danish-Swedish Farmdog | -0.08 | 0.05 | 0.079 |
| Age (standardised) * Dogue de Bordeaux | -0.12 | 0.10 | 0.248 |
| Age (standardised) * English Springer Spaniel | -0.02 | 0.05 | 0.730 |
| Age (standardised) * Finnish Lapponian Dog | 0.10 | 0.05 | 0.032 |
| Age (standardised) * Lagotto Romagnolo | 0.03 | 0.04 | 0.451 |
| Age (standardised) * Newfoundland | 0.15 | 0.09 | 0.105 |
| Age (standardised) * Portuguese Water Dog | 0.22 | 0.06 | < 0.001 |
| Age (standardised) * Rottweiler | 0.15 | 0.03 | < 0.001 |
| Age (standardised) * Samoyed | -0.14 | 0.06 | 0.012 |
| Age (standardised) * Spanish Water Dog | 0.02 | 0.06 | 0.725 |
| Age (standardised) * St. Bernard | 0.16 | 0.11 | 0.154 |
| Age (standardised) * Staffordshire Bull Terrier | <-0.01 | 0.04 | 0.938 |
| Age (standardised) * Tibetan Terrier | -0.19 | 0.09 | 0.040 |
| Age (standardised) * Welsh Springer Spaniel | -0.04 | 0.05 | 0.395 |
| Sex (female) * American Staffordshire Terrier | -0.30 | 0.13 | 0.019 |
| Sex (female) * Bernese Mountain Dog | 0.04 | 0.08 | 0.658 |
| Sex (female) * Border Collie | -0.48 | 0.10 | < 0.001 |
| Sex (female) * Boxer | -0.34 | 0.10 | 0.001 |
| Sex (female) * Bullmastiff | -0.02 | 0.245 | 0.938 |
| Sex (female) * Chow Chow | -0.41 | 0.21 | 0.047 |
| Sex (female) * Cocker Spaniel | -0.44 | 0.10 | < 0.001 |
| Sex (female) * Danish-Swedish Farmdog | 0.18 | 0.11 | 0.103 |
| Sex (female) * Dogue de Bordeaux | -0.27 | 0.23 | 0.254 |
| Sex (female) * English Springer Spaniel | -0.27 | 0.12 | 0.018 |
| Sex (female) * Finnish Lapponian Dog | -0.06 | 0.10 | 0.588 |
| Sex (female) * Lagotto Romagnolo | -0.10 | 0.09 | 0.245 |
| Sex (female) * Newfoundland | -0.20 | 0.21 | 0.339 |
| Sex (female) * Portuguese Water Dog | -0.01 | 0.15 | 0.971 |
| Sex (female) * Rottweiler | 0.01 | 0.08 | 0.901 |
| Sex (female) * Samoyed | 0.11 | 0.14 | 0.404 |
| Sex (female) * Spanish Water Dog | 0.17 | 0.13 | 0.207 |
| Sex (female) * St. Bernard | -0.77 | 0.25 | 0.002 |
| Sex (female) * Staffordshire Bull Terrier | -0.31 | 0.10 | 0.002 |
| Sex (female) * Tibetan Terrier | 0.16 | 0.21 | 0.451 |
| Sex (female) * Welsh Springer Spaniel | 0.03 | 0.11 | 0.775 |
| Year * American Staffordshire Terrier | 0.03 | 0.02 | 0.104 |
| Year * Bernese Mountain Dog | 0.03 | 0.01 | 0.017 |
| Year * Border Collie | 0.03 | 0.01 | 0.045 |
| Year * Boxer | -0.01 | 0.01 | 0.619 |
| Year * Bullmastiff | -0.05 | 0.03 | 0.137 |
| Year * Chow Chow | 0.18 | 0.03 | < 0.001 |
| Year * Cocker Spaniel | <0.01 | 0.01 | 0.912 |
| Year * Danish-Swedish Farmdog | 0.01 | 0.02 | 0.476 |
| Year * Dogue de Bordeaux | 0.03 | 0.04 | 0.396 |
| Year * English Springer Spaniel | 0.02 | 0.02 | 0.192 |
| Year * Finnish Lapponian Dog | 0.01 | 0.02 | 0.616 |
| Year * Lagotto Romagnolo | -0.02 | 0.01 | 0.247 |
| Year * Newfoundland | 0.03 | 0.03 | 0.193 |
| Year * Portuguese Water Dog | -0.03 | 0.02 | 0.239 |
| Year * Rottweiler | 0.03 | 0.01 | 0.008 |
| Year * Samoyed | 0.05 | 0.02 | 0.013 |
| Year * Spanish Water Dog | 0.01 | 0.02 | 0.671 |
| Year * St. Bernard | -0.04 | 0.04 | 0.343 |
| Year * Staffordshire Bull Terrier | -0.02 | 0.02 | 0.215 |
| Year * Tibetan Terrier | -0.02 | 0.03 | 0.600 |
| Year * Welsh Springer Spaniel | -0.02 | 0.02 | 0.274 |
